# Transient lower cranial nerve palsies following spinal anesthesia with bupivacaine-fentanyl combination for transurethral resection of the prostate

**DOI:** 10.11604/pamj.2020.35.62.4005

**Published:** 2020-03-03

**Authors:** Ali Akhaddar, Mohcine Salami, Youssef Darouassi

**Affiliations:** 1 Department of Neurosurgery, Avicenne Military Hospital, Marrakech, Morocco; 2 Department of Neurosurgery, Mohammed V Military Teaching Hospital, Rabat, Morocco; 3 University of Mohammed V Souissi, Rabat, Morocco; 4 Department of Oto-Rhino-Laryngology, Mohammed V Military Teaching Hospital, Rabat, Morocco

**Keywords:** Dysphagia, dysphonia, neurologic complication, spinal anesthesia, fentanyl, bupivacaine

## Abstract

Spinal anesthesia is a widely used regional anesthesia for many infra-umbilical surgical procedures with proven efficacy and safety. However, although rare, some neurologic complications can occur with potentially life threatening consequences. Among them, lower cranial nerve palsies have been rarely reported in the literature. We report such a case in a 75-year-old man with transient dysphagia, dysphonia and spinal accessory nerve palsy occurring four days after spinal anesthesia for transurethral resection of the prostate. His symptoms completely resolved spontaneously within 2 weeks. The possibility of lower cranial nerve palsies should be added to the potential complications during or following spinal anesthesia with bupivacaine-fentanyl combination. Although transitional, this complication may occur few days after the procedure and need to be promptly recognized, carefully evaluated and treated by conservative measures.

## Introduction

Spinal anesthesia is a widely used regional anesthesia for many obstetric, urologic, orthopedic and digestive surgical procedures with proven efficacy and safety. However, although rare, some neurologic complications can occur with potentially life threatening consequences [1–4]. To our knowledge, lower cranial nerves palsies have been reported only eleven times in the literature [1, 2, 5–9]. We report such a case with transient dysphagia, dysphonia and spinal accessory nerve palsy occurring four days after spinal anesthesia for transurethral resection of the prostate.

## Patient and observation

A 75-year-old man without medication coexisting diseases underwent transurethral resection of the prostate for a prostatic hypertrophy. He had no history of trauma, headache or coagulation abnormalities. Spinal anesthesia was conducted by puncturing the L3-L4 space and injecting 12.5 mg of hyperbaric bupivacaine (2.5 ml of 0.5% solution) combined with 25 microg of fantanyl after free flow of cerebro-spinal fluid (CSF) with the patient in the sitting position. The course of anesthesia was without incident. During surgery, excision of the prostate was conducted. His intraoperative vital signs were normal and the surgery was completed uneventfully. The operation time was 75 minutes. There were no symptoms of a postdural puncture headache but on the fourth postoperative day, the patient started to have mild occipital headache with some cervicalgia without vomiting. In addition, he developed some difficulty in swallowing and speaking with change of phonation. Initially, the patient was not too bothered by his symptoms and he was discharged from the hospital on the eighth postoperative days. Since the symptoms persisted with decreased range of shoulders movement and increased salivation, the patient presented to our department three days later. On examination, he was conscious without fever or neck stiffness. Neurologic, otorhinolaryngologic and nasofibroscopic examination revealed bilateral paresis of the ninth, tenth and eleventh cranial nerves without pyramidal or cerebellar signs. Cranial computed tomography scan was performed and did not show any abnormalities. The patient was treated conservatively with analgesia and diet modification. His symptoms resolved progressively after about 2 weeks of onset without any residual neurological deficit. At the end of the first month postoperatively, the patient had no subjective symptoms.

## Discussion

There are several reports in the literature of acute neurological complications after spinal anesthesia including altered level of consciousness, aphasia, hemiparesis and palsy of occulomotor, trigeminal and facial nerves [2–4, 10]. However, lower cranial nerve palsies have been rarely reported in the literature. In our review, only 11 cases were found, of which all were young women (between 19 and 32 year-old) during spinal anesthesia for obstetric procedures (8 cases for labor analgesia and 3 cases for cesarean section) [1, 2, 5–9]. All cases reported in English literature were summarized in Table 1. Our patient is the first man reported and older than those previously described. Several authors trend to explain this rare phenomenon. They hypothesized that the reaction was caused by cephalad spread of either the opioid or the bupivacaine (occurrence of high sensory blockade), toxic reaction of these drugs or subdural catheter placement (than the subarachnoid space) [2, 5, 7, 9].

**Table 1:** Summary of the 11 cases with lower cranial nerves complications following spinal anesthesia reported in the literature

Author [ref], year	Sex, age	Procedure	Intrathecal drugs	Neurologic complication
Cohen [1], 1993	F, 28 y	Labor analgesia	Sufentanil 10 mcg	Dysphagia, difficulty in taking deep breath and facial numbness
Hamilton [5], 1995	F, 26 y	Labor analgesia	Sufentanil 10 mcg	Dysphagia, itching on face
Hamilton [5], 1995	F, 32 y	Labor analgesia	Sufentanil 10 mcg + bupivacaine	Dysphagia, facial and upper limb numbness
Hamilton [5], 1995	F, 20 y	Labor analgesia	Sufentanil 10 mcg	Dysphagia, dry throat
Currier [6], 1997	F, 21 y	Labor analgesia	Fentanyl 20 mcg	Dysphagia and inability to clear throat
Currier [6], 1997	F, 27 y	Labor analgesia	Fentanyl 25 mcg + bupivacaine 2.5 mg	Generalized itching, dysphagia, tingling around lips and fingertips
Musch [7], 1999	F, 30 y	Cesarean section	Anesthetic-opioid	Dysphagia
Kuczkowki [8], 2003	F, 21 y	Labor analgesia	Fentanyl 10 mcg + bupivacaine 2.5 mg	Dysphagia and inability to talk
Smiley [9], 2007	F, 23 y	Labor analgesia	Fentanyl 20 mcg + bupivacaine 2.5 mg	Dysphagia and loss of gag reflex
Smiley [9], 2007	F, 19 y	Cesarean section	Fentanyl 20 mcg + bupivacaine 12 mg	Dysphagia and loss of gag reflex
Ray [2], 2012	F, 31 y	Cesarean section	Fentanyl 25 mcg + bupivacaine 7.5 mg	Aphonia and facial tingling
Present case, 2014	M, 75 y	Transurethral prostate resection	Fentanyl 25 mcg + bupivacaine 12.5 mg	Dysphagia, dysphonia and spinal accessory nerve palsy

F: female; M: male; y: years; mg: milligrams; mcg: micrograms

In all previous reported cases, all the symptoms started shortly (few minutes) after intrathecal injection of drugs (opioid alone or combined with bupivacaine) and disappeared within less than 90 minutes. Our literature search revealed no other reports of this type of lower cranial nerve paresis including spinal accessory nerve palsy occurring four days after spinal anesthesia. It is well known that in old patients as in our case, compression of the thecal sac, spinal canal abnormalities and difficulties with block placement are risk factors for total spinal block or accidental subdural injection. Lower cranial nerve palsies may occur as a result of pneumocephalus or posterior fossa hemorrhage, but in our case, cranial CT-scan was normal. This late transient complication is difficult to explain in our patient but as reported by Fang and colleagues, we speculate that CSF depletion (decrease of CSF pressure) and intracranial hypotension may play a role. It has been explained on the basis of CSF loss causing descent of the brain and stretching of the nerves [10]. Our patient suffers considerable anxiety. He was managed conservatively and fortunately his symptoms resolved progressively and completely without any residual neurological deficit.

## Conclusion

The possibility of lower cranial nerve palsies should be added to the possible side effects of bupivacaine-fentanyl combination used in patients during and following spinal anesthesia. Although transitional, this complication may occur few days after the procedure and need to be promptly recognized, carefully evaluated and treated by conservative measures.

## Competing interests

The authors declare no competing interests.
